# Linkages between pelagic and benthic biota in a deteriorated coastal lake after restoration, Maruit, Egypt

**DOI:** 10.1007/s10661-023-11525-x

**Published:** 2023-07-05

**Authors:** Hanan Mitwally, Hoda El Rashidy, Paul Montagna

**Affiliations:** 1 Oceanography Department, Faculty of Science, Alexandria University, Alexandria, Egypt; 2 Harte Research Institute, Texas A&M University-Corpus Christi, Corpus Christi, TX USA

**Keywords:** Ectoparasites, Fish, Meiofauna, Food web, Halacaridae, Passive movement, Organic matter

## Abstract

Until the 1960s, Lake Maruit was one of Egypt’s most productive coastal brackish lakes. Continuous polluted discharge from Alexandria city resulted in long-term deterioration. The Egyptian government started a lake restoration program in 2010. Biological linkages between pelagic and benthic communities were assessed in November 2012 using parasitism and predation. This study examined ectoparasites infesting tilapia fish from 300 samples. The platyhelminth ectoparasite, Monogenea, and parasitic-copepod *Ergasilus lizae* were detected. Platyhelminthes parasitized *Oreochromis niloticus* and *Oreochromis aureus*, whereas the crustacean parasitized *Coptodon zillii*. The parasitic prevalence was low for *Cichlidogyrus* sp. and *Ergasilus lizae*. Benthic biotas were similar across basins. Fish abundance does not respond directly to benthic biotic components. Phytoplankton and benthic microalgae were not the main fish diet. Data on Halacaridae and fish clustered, indicating that either Halacaridae responds to their environment like fish or fish prey upon them because of their size. Linear correlations between pelagic, benthic biota, and parasite-infected fish indicate parasites may control their hosts. Some bioindicators indicate that stressed ecosystems differ from unstressed ecosystems. Fish species and biota abundances were low. Inconsistency in the food web and an absence of direct interactions between prey and predators are bioindicators of disturbed ecosystems. The low prevalence of ectoparasites and lack of heterogenous distribution of the various examined biota are bioindicators of habitat rehabilitation. Ongoing biomonitoring to better understand habitat rehabilitation is suggested.

## Introduction

Coastal lakes are productive water bodies (Kjerfve, 1994) that suffer from natural disturbances and anthropogenic stressors that cause significant deterioration of their habitats worldwide (Kennish & Paerl, 2010). Restoration of physical, chemical, and biological components of deteriorated aquatic ecosystems is the aim to improve water quality and reduce eutrophication (Søndergaard, 2007) or to control allochthonous inputs or restore the natural hydrology (Janssen et al., 2019). In contrast to natural recovery, some restoration methods, such as hydrological, mechanical, and dredging, may harm biota or have other negative effects on the environment (Alhamarna & Tandyrak, 2021; Amico et al., 2004; Wilcox' & Whillans, 1999).

Pelagic and benthic habitats are essential components of lakes (Schindler & Scheuerell, 2002), and each includes essential flora and fauna that differ in mode of life and dispersion. The pelagic–benthic linkages are complicated because of many direct and indirect intermediate relationships (Palmer et al., 2000). Predation is one direct influence of the pelagic biota that affects benthic species, which act as intermediate hosts for many parasitic species and are responsible for parasitic transmission to the food web via vertical migration to the hyper-benthic zone or the water column (Amundsen et al., 2013; Marcogliese, 2002). In shallow lakes, the ecological interaction strengths of the benthic–pelagic linkages increase as the lake depth and surface area decrease (Schindler & Scheuerell, 2002), and their study is mandatory to understand some environmental issues in aquatic habitats (Lamberti et al., 2010). The high fish production in a shallow lake is partly related to benthic organisms that represent a food source. Several fish and benthos species are directly linked with parasites as their host, whereas the latter relies on host species for feeding (Hechinger et al., 2006). Fish parasites were used to assess environmental changes in coastal waters as early warning indicators (Kleinertz & Palm, 2013; Palm, 2011). Some studies applied the parasite index in an association with health assessment index as an indicator for water quality (Crafford & Avenant-Oldewage, 2009). The highest ectoparasite infestation in some marine ecosystems is found in the most polluted areas (Vidal, 2008), the ectoparasite index is a quick and simple way to assess the health of a fish community (Sara et al., 2013), and parasite effects on fish health are recommended for monitoring in stream management programs (Nedić et al., 2018).

Most lake ecosystems have a high amount of allochthonous organic matter exceeding the autochthonous amounts, and bacterial activity can mobilize external organic matter into the food web (Kufel et al., 1997; Wetzel, 1992). Allochthonous organic matter can have a relatively high contribution (60%) to fish biomass production and most of their potential prey (Karlsson et al., 2015). But, allochthonous matter can reduce the penetration of light, which can suppress food web productivity (Craig et al., 2015). However, the source of organic matter (e.g., terrestrial or detritus-based) can affect direct and indirect use by different trophic levels and different feeding modes of invertebrates (Riera, 1998).

Lake Maruit is a brackish ecosystem located south of Alexandria, Egypt, has a small surface area and is separated from the sea by high carbonate ridges formed during the Pleistocene age (El- Masry & Friedman, 2000). It has an artificial connection with the sea via the El-Mex pump station that maintains the water depth at ~ 2.8 m to 3 m below sea level. Since the 1960s, it was a sink for different pollutants and agricultural effluents discharged from the Qalaa and El-Umum drains (El-Rayis et al., 2019). Lake Maruit’s surface area had gradually reduced to 63 km^2^ (Ahmed & Barale, 2014), its physical and biological conditions changed, and its water quality was effected by discharges and land reclamation projects (El Kafrawy et al., 2017). It was designated in 1992 as the most polluted lake in Egypt (EEAA, 2015).

Many studies have documented the long-term deterioration of water quality and heavy metal impacts on different biota prior to the restoration program (1969–2010, Table 1) (Abd-Allah & Ali, 1994; Abdallah, 2011; Adham et al., 2008; Amr et al., 2005; Arafa & Ali, 2008; El-Rayis et al., 2019; El Nabawi et al., 1987; Elghobashy et al., 2001; Hassaan et al., 2009; Hassan & El-Rayis, 2018; Hussein & Gharib, 2012; Khalil, 1998; Khalil & Koussa, 2013a, b; Oczkowski & Nixon, 2008; Prenner et al., 2006; Saad, 1973). Adverse effects are caused by hydrocarbon pollution and pesticides (Barakat et al., 2002, 2010, 2012; Khairy, 2013). However, studies after lake restoration (2011–2020) reported inconsistent results concerning water quality, eutrophication, and effects on biota (i.e., plankton, benthos, and fish) (Table 1). Few studies focused on the socioeconomic impacts (Abdrabo & Hassaan, 2010), and many studies proposed additional lake restoration projects (El-Hattab, 2015; El-Rayis et al., 2019; Selim & El-Raey, 2018). The restoration program started when the government dug channels in 2010 diverting the El-Qalaa drain’s discharge directly into the El-Umum drain without passing through the Main Basin. In 2012, another channel was dug to divert the Western wastewater plant (WWWPT) effluent into the El-Mex pump station via the El-Umum drain (Shaaban, 2022; Shreadah et al., 2020).

**Table 1:** Documented long-term deterioration of Lake Maruit

Decade/year of study	Water quality/ecological parameter	Biological domain	Reference
1969–1970	Phosphate content	Variable consumption by phytoplankton	(Saad, 1973)
1985	Heavy metals in sediments	Tilapia muscles	(El Nabawi et al., 1987)
1990–1991	Limnological analysis	Chlorophyll and pH relations	(Massoud & Safty, 2004)
1992	Organochlorine compounds	Tilapia species muscles	(Abd‐Allah &Ali, 1994)
1992–1993	Chemical pollution	Tilapia physiological response	(Adham et al., 2008)
1993–2000	Nutrient enrichment	Fish caught tilapia species	(Oczkowski & Nixon, 2008)
1996–1997	Organic pollution	Eutrophication, legal limits, fish muscles	(Khalil, 1998)
1998–1999	––	Macrofauna, gastropods	(Khalil & Koussa, 2013b)
1998–1999	––	Zooplankton, rotifers	(Khalil & Koussa, 2013a)
2000	Water quality and EEAA law (4/1994)	Cadmium, lead, and fish flesh	(Amr et al., 2005)
2000	Heavy metals water and sediments	Bioaccumulation tilapia fish	(Elghobashy et al., 2001)
2001	Sediment trace-metals	Insects bioassay	(Prenner et al., 2006)
2003–2004	––	Phytoplankton Bacillariophyceae	(Hussein & Gharib, 2012)
2005	Polycyclic aromatic hydrocarbons	––	(Barakat et al., 2010)
2006–2007	The pH and dissolved oxygen	The pH, aquaculture, and fish farm	(El-Rayis et al., 2019)
2007	Water quality	Aquaculture	(Hassaan et al., 2009)
2007 and 2014	Engineered waterworks	Biological restoration	(Selim & El-Raey, 2018)
2007–2008	Anoxic and oxic condition	––	(Hassaan et al., 2017)
2007–2008	H_2_S, oxygen, cobber, and manganese	Anaerobic bacteria	(Hassan & El-Rayis, 2018)
2008	Heavy metals	Musculature biochemical tilapia	(Arafa & Ali, 2008)
2009	Sediments phosphorus	Eutrophication	(Abdallah, 2011)
2009	Sediments phenolic compounds	––	(Khairy, 2013)
2010	Socioeconomic effect	Impact flora, fauna, and fish production	(Abdrabo & Hassaan, 2010)
2011	Heavy metals	Affect biota life	(Mohammed & Kondrashin, 2013)
2012	Oxygen, phosphorus	Meiofauna, macrofauna	(Mitwally, 2015)
March 1991, August 1995, May 2004, and May 2013	Lake restoration scenarios	––	(El-Hattab, 2015)
2013	Oxygen, BOD, and ammonia	Macrofauna, Mollusca	(Khalil et al., 2016)
2013–2014	Dissolved oxygen, phosphorus, and nitrogen	High eutrophic conditions	(Shreadah et al., 2020)
2014	Environmental features	Vegetation patterns	(Ahmed & Barale, 2014)
2014	Dissolved oxygen, phosphate, trace metals	Macrophytes	(Tadros et al., 2019)
2014	Dissolved oxygen phosphate	Chlorophyll-a	(El Zokm et al., 2018)
2014–2015	Phosphate, nitrate/phosphate ratio, and ammonia	Eutrophication	(Abdelfattah et al., 2017)
2015	––	Phytoplankton Bacillariophyceae	(Abdel-Hamid & Galal, 2019)
2016	Dissolved oxygen, nutrient, phosphorus, and heavy metals	Chlorophyll-a, total coliform bacteria, and *E. coli*	(Abd El-alkhoris et al., 2020)
1972–2016	Satellite images, 20% reduction of lake surface area	Aquatic vegetation	(El Kafrawy et al., 2017)
2016–2017	Ammonia, nitrite, nitrate, and BOD	Algal blooming and eutrophication	(Helal et al., 2017)
2018	Temperature, salinity, dissolved oxygen, pH, hydrogen sulfide, and nutrients salts	Chlorophyll-a	(Shaaban, 2022)

By comparing stations within lake basins, the present study assessed Lake Maruit after two phases of the restoration program. Parasitism infections are directly related to pollution and could indicate ecosystem water quality (Jiménez-García et al., 2020). So, the first objective was to assess Lake Maruit’s health by investigating the prevalence of fish ectoparasites; second, to compare biotas’ variability (fish numbers, meiofaunal groups, benthic microalgae, microbes, and phytoplankton) among stations within basins; and third, to discover trophic links in the food web and associate organic matter with pelagic and benthic biota. Disturbance can affect community structure trophic links in the grazing or detritus food chains (Vinogradov et al., 2018).

## Materials and methods

**Fig. 1:**
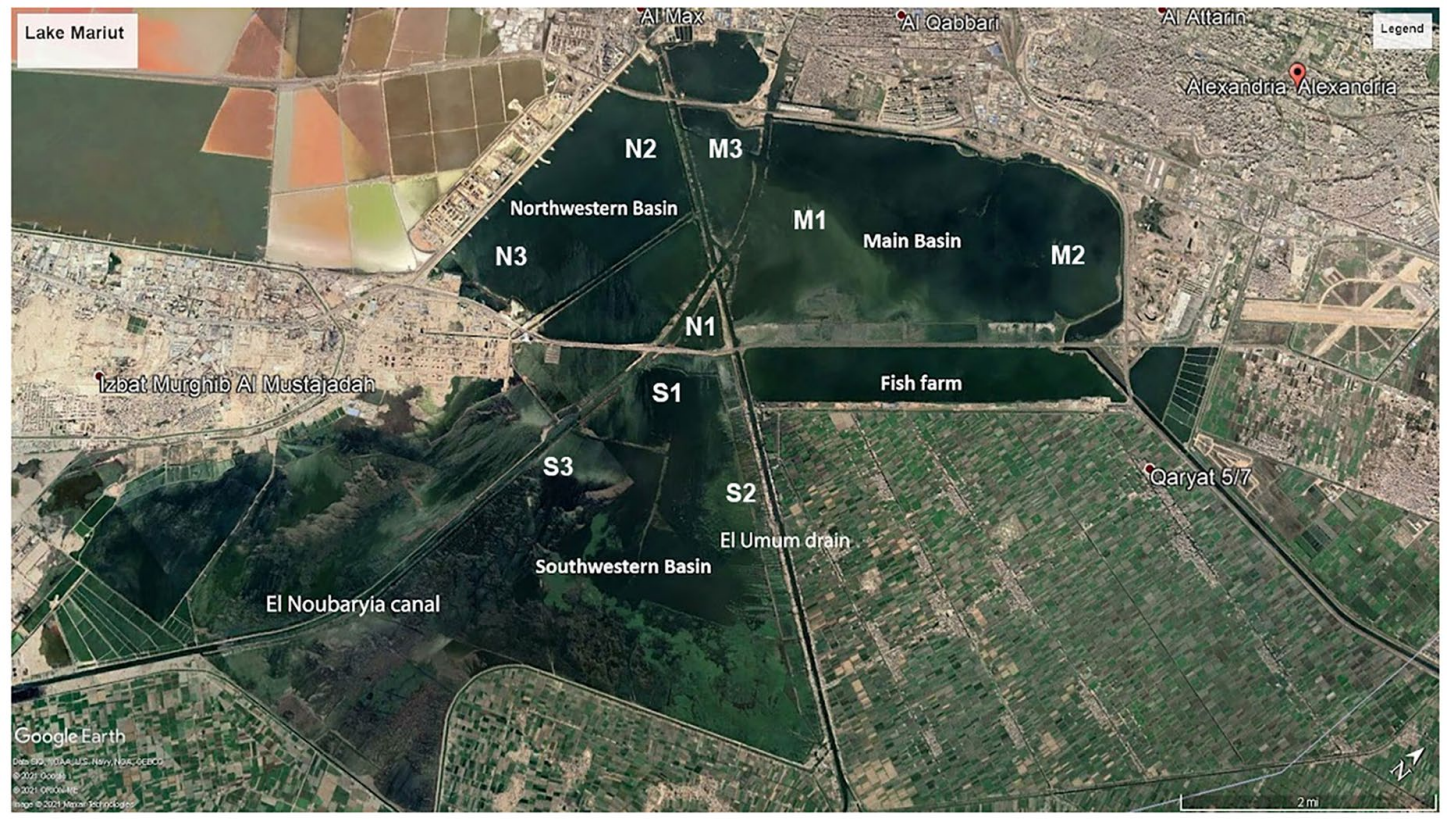
Map of basins and stations in Lake Maruit, Egypt. Abbreviation: M, Main Basin; N, Northwestern Basin; S, Southwestern Basin. This is a google earth recent downloaded map for Lake Maruit

Lake Maruit extends about 25 km south of Alexandria, with a maximum width of 10 km and an average depth of one meter (Abdel-Moneim et al., 2012; Barakat et al., 2010). The lake is divided into five basins: Main Basin, the Southeast Basin, the Northwest Basin, the Southwest Basin, and the Fish Farm Basin. Three water sources feed the lake daily: (1) the main agricultural drain, El-Umum, which is connected to the M on its west side via many channels; (2) the El Qalaa drain that feeds the lake with a mixture of agricultural and sewage effluents from the East Wastewater Plant (EWWTP) (Shreadah et al., 2020); and (3) the West Wastewater Plant (WWWTP) on the southwest side of the M. The different discharge sources mix with freshwater discharge from the El Noubaryia canal, and industrial oil effluents enter from the N from the oil company at Main basin, which are transported into the Mediterranean Sea via the El-Mex pump station (Fig. 1). Sources of dumped pollution into the S are limited, and this basin is bounded by El-Umum agricultural drain and El Noubaryia freshwater canal.

### Sampling sites and study design

Three stations were sampled in three basins: M, N, and S (Fig. 1). Three grab deployments were collected at each station, and three subsamples were taken from each grab deployment. Due to the shallowness and high contamination levels of Lake Maruit, stations were sampled randomly to cover most of the area in each basin. At all basins, station 1 was near the freshwater canal. Stations M2 and M3 were near WWWTPs and El-Umum drains. Stations N2 and N3 are located next to El-Umum agricultural drain and oil companies, whereas stations S2 and S3 are situated between El-Umum drain and El Noubaryia canal. The study design is hierarchical, where the basin is a fixed factor, stations are nested within basins, and deployments are nested within stations. It is a fully nested design where the correct *F*-test for the basin is the mean square (MS) for the basin divided by the MS for the station, and for the station, its MS is divided by the MS for deployment. In this study, 81 samples were collected for 3 basins multiplied by 3 stations, 3 deployments, and 3 replications.

#### Field collection

All samples were collected in November 2012, after two years of the restoration program initiation. Fish were caught with trammel nets using watershed police boat (zodiac). Sediment samples were collected using small Van Veen grab size (9 kg). Five sediment samples were taken from each grab. For meiofaunal analysis, three subsamples were taken using an 11-cm hand-held core with a 4.9-cm^2^ surface area. For benthic microalgae (BMA) and total organic matter (TOM) analyses, two samples were withdrawn by a small plastic vial (a surface area of 2 cm^2^). Sediments for microbial analysis were collected using a 1-ml sterilized syringe and stored in sterilized bags. Sediment samples were kept in an icebox for laboratory analysis within 3 to 48 h after collection. Three liters of water samples were collected by Niskin bottle for phytoplankton analysis, and a neutral 4% formalin solution with a few drops of acidified Lugol’s solution was used for fixation (Montagnes et al., 1994).

### Laboratory analysis

Fish samples were sorted and identified to species level, total lengths were measured to the nearest (cm), and fish were grouped into size classes. The gills of each fish were examined for ectoparasites; and parasites were collected from the infected gill filaments, preserved in 70% ethyl alcohol, inspected, and identified on an Olympus compound microscope (Kabata, 1970).

The Huys et al. (1996) decantation method was used to extract meiofauna from sediment samples. The sample was vigorously agitated in water in a 1-l stoppered container, left to stand for a few seconds allowing sediment particles to settle, and the supernatant water was poured through a 63-μm mesh sieve. The procedure was repeated several times to remove all organisms. Organisms were sorted and counted, and abundances were extrapolated to individuals per 10 cm^2^ (Mitwally, 2015).

Phytoplankton samples were left for settlement for 36 to 48 h, and then, the supernatant was siphoned, the remaining volume adjusted to a 100 ml, cells were counted using an inverted microscope, and abundances were expressed as cells/l. Benthic microalgae were extracted, counted according to Schwinghamer (1983), and reported as cell/cm^3^ of sediment.

For microbial analysis, sediment samples were diluted in sterile water (aged and filtered through a 0.45-μm filter), spread plated onto nutrient agar medium (beef extract 3 g/l, peptone 5 g/l, and agar 15 g/l) (Guerra-Floresa et al., 1997), sonicated for 5 min, vigorously shaken, and allowed to settle for 5 s, and the collected supernatant water was incubated at 30 °C for a period of 24 to 48 h before counting colony-forming units (CFU/ ml) (Chelossi et al., 2003).

The TOM was analyzed according to El Wakeel and Riley (1957). A dried-ground sediment sample was treated with chromic acid and titrated with ferrous ammonium sulfate solution, using the ferrous phenanthroline indicator. The percentage of TOM was calculated using organic carbon values in the Olausson (1975) equation.

### Data analysis

The biota and TOM data were square-root transformed. The Durbin–Watson test (Durbin & Watson, 1971) was used to check for autocorrelation between the four response variables (number of total fish, non-infected fish, infected fish with ectoparasites, and the ratio of infected to total fish number). The multicollinearity among ten biological variables and TOM was examined using Draftsman correlations (Anderson et al., 2008). The fully nested ANOVA was run to test for differences in the mean data of each response (fish) and predictors (10 different biotas and TOM) among basins and stations. In many cases, the basin factor was not significant, but the station factor was significant. This required the experimental design to devolve to a 1-way, simple main effects, model for basin–station differences, and grab deployments within each station are a nested from replication. Nested ANOVA and simple main effects ANOVA were run using SAS software. The Tukey post hoc test was applied after a significant *F* ratio to test for pairwise comparisons among level means, whereas the *t*-test was used to determine if the length of infected fish differed from the non-infected individuals using Minitab software. Multiple linear regression analysis (GLMM) was applied four times to predict linear relationships between each response variable of fish data and the biotic predictors as a proposed fish diet at the significant – level ≤ 0.05.

All biota community structures were examined with multivariate analyses using PRIMER 7 software. The first step was constructing a Bray–Curtis similarity index of square-root transformed data. Data was plotted using nonmetric multidimensional scaling (*n*MDS). The PERMANOVA procedure (Anderson et al., 2008) was run to test for differences in community structure among basins, stations nested in basins, and deployments nested in basins and stations (i.e., a fully nested model). The hierarchical agglomerative cluster analysis based on the association index (Clarke & Gorley, 2015) was used to examine links between pelagic biota, benthos, TOM, and their linkage to fish. The test statistic (Pi) was used to identify the deviation of the observed from the predicted permuted profiles (Clarke et al., 2008) at the significant *α* level of ≤ 0.05. Coupling between the individual clusters of the same trophic level was classified as a tie, whereas a “linkage” was classified as different trophic level couplings. The *n*MDS analysis was conducted twice: to visualize the distances between pelagic and sediment biota and to discriminate among the lake basins. The test was based on the Bray–Curtis similarity matrix and the same cluster analysis. The similarity percentage analysis (SIMPER) is using the same Bray–Curtis matrix at a lower cut-off index of 70% (Clarke & Gorley, 2015) to test for the percentage of similarity among the lake basins and station groups and to identify organisms that had contributions across lake basins and stations. Stations were classified into three groups: Gr. 1 includes stations 1 across all basins, and Gr.2 and Gr. 3 each include all stations 2 and 3, respectively.

## Results

**Fig. 2:**
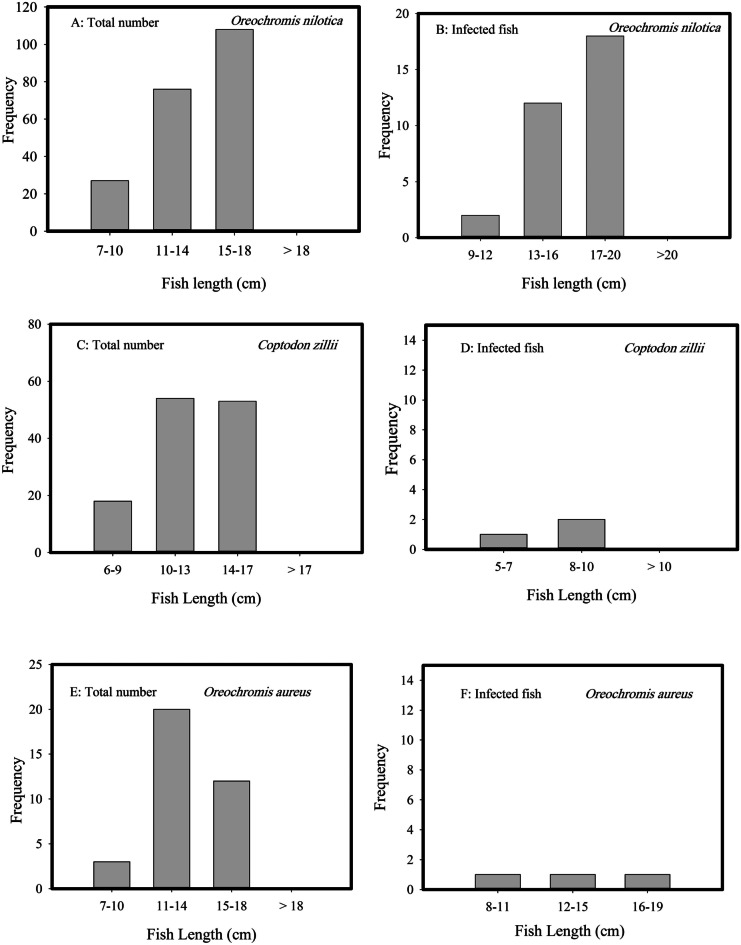
Frequency length classes for total fish number per species and the infected individuals

There were three tilapia fish species, *Oreochromis niloticus* (Linnaeus, 1758), *O. aureus* (Steindachner, 1864), and *Coptodon zillii* (Gervais, 1848), but *O. niloticus* was the dominant species. Each fish species grouped into three size classes (Fig. 2). *O. niloticus* was the longest (10–20 cm), and the smallest was *C. zillii* (6–17 cm). Among parasite-infected fish, *O. niloticus* was the longest (17–20 cm) and *C. zillii* was the shortest (5–10 cm). The parasitic infestation was distributed evenly among size categories of *O. aureus*. There were no statistically significant differences in fish size between the infected and non-infected fish (*t*-test, *P* = 0.85). The ectoparasites comprised the parasitic Monogenea (*Cichlidogyrus* sp.) and parasitic copepods (*Ergasilus lizae*). *Cichlidogyrus* sp. infected the two *Oreochromis* species, and *Ergasilus lizae* parasitized *C. zillii*. The prevalence of infections of Monogenea and parasitic copepods was 15% and 3%, respectively.

The biota predictors were tabulated by basin in Table 2. The highest and the lowest abundances (individuals 10 cm^−2^) of total meiofauna and its taxa were detected at the N and S basins, respectively, with few exceptions (e.g., Halacaridae). The M basin contained the highest abundances of phytoplankton (78.02 × 10^3^ ± 32.83 × 10^3^ cell/l), BMA (32.70 × 10^3^ ± 27.47 × 10^3^ cell/l), and microbes (228.1 ± 119.7 CFU/ml). The lowest phytoplankton and microbe abundances were detected at S basin (3.19 × 10^3^ ± 1.7 10^3^ cell/l, 167.4 ± 60.9 CFU/ml, respectively), whereas the lowest density of BMA was recorded at N basin. The percentage of TOM ranged from 8.5% at M to 4.8% at S basins.

**Table 2:** Summary of biota and percentage of total organic matter (TOM%) among Lake Maruit basins during November 2012 (mean ± standard deviation)

Variables	M		S		N	
	Mean	± SD	Mean	± SD	Mean	± SD
Total meiofauna (individuals 10 cm^−2^)	1088.2	621.3	1046.8	1156.2	2830.8	2409.1
Nematodes (individuals 10 cm^−2^)	579.8	420.6	647.7	861.5	1577.3	1392.0
Harpacticoida (individuals 10 cm^−2^)	278.6	175.4	176.6	166.9	441.7	352.1
Foraminifera (individuals 10 cm^−2^)	116.0	65.6	97.6	77.2	420.9	453.1
Ostracods (individuals 10 cm^−2^)	65.0	52.6	84.0	80.0	286.3	301.9
Turbellaria (individuals 10 cm^−2^)	42.8	40.1	38.7	36.9	100.8	113.8
Halacaridae (individuals 10 cm^−2^)	6.0	5.8	2.2	3.4	3.7	4.6
Phytoplankton (cell 10^3^/l)	78.02	32.83	3.19	1.7	17.85	9.62
Benthic microalgae (cell 10^3^/cm^3^)	32.70	27.47	13.85	14.37	6.24	1.25
Microbial counts (CFU/ml)	228.1	119.7	167.4	138.7	191.3	60.9
TOM (%)	8.5	1.4	4.8	2.0	6.7	0.5

*TOM* total organic matter, *M* Main Basin, *S* Southwestern Basin, *N* Northwestern Basin

The Durbin–Watson statistics revealed positive autocorrelations among the fish variables and ranged from 1.80 (ratio of the infected fish number to the total fish numbers) to 1.01 (the non-infected fish number). The correlation coefficients were less than the cut-off value (0.95) for all the biotic metrics and TOM, so the results are not reported here.

**Fig. 3:**
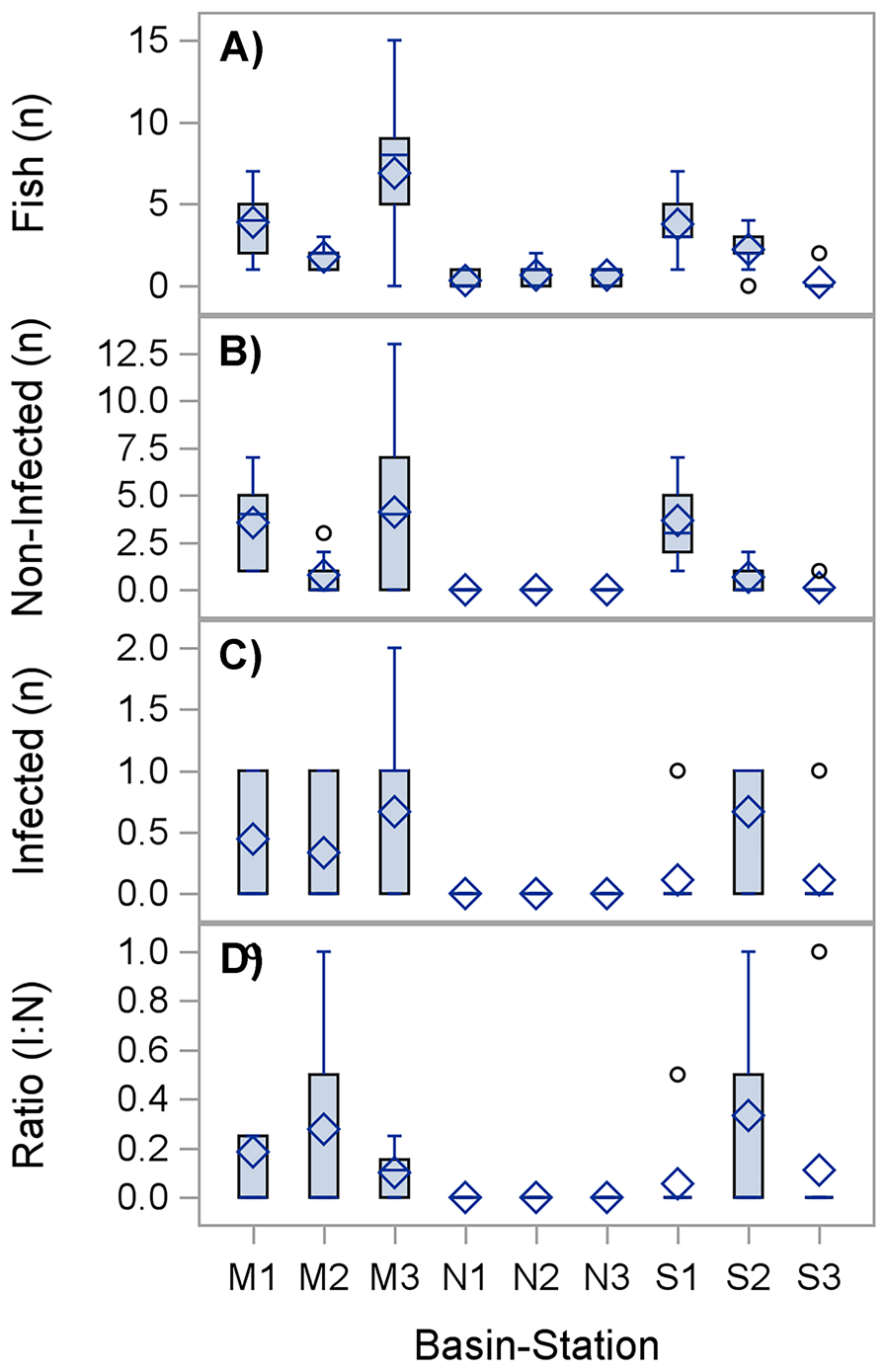
Box plots of fish metrics by stations within basins. **A **Total fish counts. **B** Non-infected fish counts. **C** Infected fish counts. **D** Ratio of the infected to the total fish counts. Abbreviations: I: N, infected to non-infected ratio; M, Main Basin; S, Southwest Basin; N, Northwest Basin

The hierarchical ANOVA revealed non-significant differences among basins in the mean numbers of four fish variables. However, the simple main effects ANOVA on stations nested in basins were significant for fish groups, except for the ratio between the infected fish to the total fish numbers (Table 3). The variations in the total fish and the non-infected fish counts were obvious in stations 1, 2, and 3 at the M and S stations (Fig. 3A, B), and the highest counts were detected within station 2 (M). Similar counts of infected fish were recorded within the M and S stations, except for St.3 (M), which had a higher standard deviation (Fig. 3C). The highest ratios were found at station 2 in the M and S (Fig. 3D). However, a simple main effect ANOVA revealed no variation among stations for the ratio data.

**Table 3:** Results of the simple main effects ANOVA based on square-root transformed data of total numbers of fish, non-infected numbers, infected fish with ectoparasites, and ratios within stations nested in basins (Station, St (Basin, Ba)) and deployment nested in stations and basins (Dep. (Ba-St.))

Group	Factor	df	SS	MS	*F* ratio	***P***	*R*^2^	CV	Mean
Total fish	St (Ba)	8	339.33	42.42	10.34	**< 0.0001**	0.556	84.38	2.4
	Dep. (Ba-St.)	15	49.83	3.32	0.77	0.7057			
	Error	51	220.83	4.33					
	Total	74	610						
Non-infected fish	St (Ba)	8	217.03	27.13	6.45	**< 0.0001**	0.44	132.59	1.55
	Dep. (Ba-St.)	15	124.06	8.27	2.75	**0.0037**			
	Error	51	153.50	3.01					
	Total	74	494.59						
Infected fish	St (Ba)	8	5.12	0.64	3.52	**0.0019**	0.299	152.28	0.28
	Dep.(Ba-St.)	15	3.33	0.22	1.31	0.2323			
	Error	51	8.67	0.17					
	Total	74	17.12						
Ratio infected/total counts	St (Ba)	8	1.01	0.13	1.64	0.1316	0.165	217.76	0.13
	Dep.(Ba-St.)	15	1.12	0.07	0.96	0.5102			
	Error	51	3.98	0.08					
	Total	74	6.11						

The bold values indicate the results ≤ 0.05

*df* degree of freedom, *SS* sum squares, *MS* mean square, *F-ratio* MS (Basin)/MS (station), *P* probability α level, *R*^*2*^* R*-square, *CV* data covariance, *Mean* overall mean of fish group values

Total meiofaunal abundance and its taxa groups were significantly different among stations nested in basins (Table 4). The deployment effect was not significant except for Ostracoda (*P* ≤ 0.0001). The other predictors (phytoplankton, BMA, microbial counts, and TOM) varied significantly among basins for phytoplankton and TOM.

**Table 4:** Results of the simple main effects ANOVA based on square-root transformed data of meiofauna and its dominant groups within stations nested in basins (station (Basin)) and deployments nested in stations and basins (Dep. (Ba-St), and the other biota variables (phytoplankton, benthic microalgae, and microbes) and TOM% among the fixed effects (Basin)

Group	Factor	df	SS	MS	*F* ratio	***P***	*R*^2^	CV	Mean
Meiofauna	St (Ba)	8	5,384,987	673123	15.72	**< 0.0001**	70%	71.42	318.6
	Dep. (Ba-St.)	16	685,301	42831	0.83	0.6499			
	Error	50	2,588,812	51776					
	Total	74	8,659,100						
Nematodes	St (Ba)	8	1,630,682	203835	9.77	**< 0.0001**	61%	87.06	180.32
	Dep. (Ba-St.)	16	333,670	20854	0.85	0.63			
	Error	50	1,232,367	24647					
	Total	74	3,196,718						
Harpacticoida	St (Ba)	8	113,552	14194	9.78	**< 0.0001**	68%	61.29	58.68
	Dep.(Ba-St.)	16	23,218	1451	1.12	0.3619			
	Error	50	64,678	1294					
	Total	74	201,448						
Foraminifera	St (Ba)	8	148,986	18623	11.06	**< 0.0001**	72%	93.15	39.75
	Dep.(Ba-St.)	16	26,947	1684	1.23	0.2804			
	Error	50	68,533	1371					
	Total	74	244,466						
Ostracods	St (Ba)	8	82,511	10314	9.25	**< 0.0001**	88%	61.13	27.31
	Dep.(Ba-St.)	16	17,845	1115	4	**< 0.0001**			
	Error	50	13,934	279					
	Total	74	114,290						
Turbellaria	St (Ba)	8	7810	976	5.42	**0.002**	66%	89.95	11.75
	Dep.(Ba-St.)	16	2882	180	1.61	0.0998			
	Error	50	5582	112					
	Total	74	16,274						
Halacaridae	St (Ba)	8	17.83	2.23	3.09	**0.0262**	39%	117.93	0.81
	Dep.(Ba-St.)	16	11.56	0.72	0.79	0.6943			
	Error	50	46	0.92					
	Total	74	75.39						
Phytoplankton	Basin	2	15,860.42	7930.21	14.35	**0.0052**	82.71%	61.33	0.0383
	Error	6	3316.06	552.6767					
	Total	8	19,176.48						
Benthic microalgae	Basin	2	1627.08	813.5376	2.11	0.2019	41.34%	107.45	0.0183
	Error	6	2309.28	384.8679					
	Total	8	3936.23						
Microbial counts	Basin	2	52,248	26,124.11	3.76	0.0874	55.62%	47.69	174.78
	Error	6	41,681	6946.89					
	Total	8	93,930						
TOM	Basin	2	27.7	13.85	9.49	**0.0138**	75.99%	17.1	7.06
	Error	6	8.75	1.46					
	Total	8	36.45						

The SS, MS, and mean of phytoplankton and benthic microalgae are divided by 10^6^

The bold values indicate the results ≤ 0.05

*df* degree of freedom, *SS* sum squares, *MS* mean square, *F-ratio* MS (Basin)/MS (station), *P* probability *α* level, *R*^*2*^* R*-square, *CV* data covariance, *Mean* mean of examined biota

PERMANOVA (Table 5) revealed significant differences for biota predictors and TOM among basins (*P*_perm_ = 0.0332), stations nested in basins (*P*_perm_ ≤ 0.0001), and deployments nested within basins and stations (*P*_perm_ = 0.0073). The pair-wise comparisons revealed noticeable differences for biota within stations nested at the M (*P*_perm_ ≤ 0.0001). S stations were significantly different, as *P*_(MC)_ revealed. At the N stations, significant variations were detected between station 1 compared to stations 2 and 3 (*P*_(MC)_ = 0.0002 and 0.0036, respectively, for station 1 vs. station 2 and station 3).

**Table 5:** PERMANOVA results and pair-wise comparisons based on the Bray–Curtis similarity of the biota abundances listed in Table 3 and the % TOM among basins and stations in basins, and within deployments nested in basins–stations at significant *α* level ≤ 0.05

Source	df	SS	MS	Pseudo-*F* statistic	*P*_perm_	Perms	*P*_(MC)_
Basin	2	30,821	15,410	4.1287	**0.0332**	9947	**0.0369**
Station (Basin)	6	23,883	3980.5	28.543	**0.0001**	9933	**0.0001**
Dep. (St-Ba)	15	2059.6	137.31	2.595	**0.0073**	9926	**0.0092**
Residuals	51	2698.5	52.912				
Total	74	60,663					

Pair-wise comparisons within Station (Basin)							
Factor	Group		t	*P*_Perm_	Perms		*P*_(MC)_
Station (Basin)	St.1, St.2		45.766	0.0984	10		**0.0001**
M	St.1, St.3		6.2821	**0.0001**	10		**0.0016**
	St.2, St.3		5.4886	**0.0001**	10		**0.007**
Station (Basin)	St.1, St.2		3.3518	0.0992	10		**0.021**
S	St.1, St.3		17.071	0.0977	10		**0.0003**
	St.2, St.3		3.6062	0.0991	10		**0.0078**
Station (Basin)	St.1, St.2		9.1468	0.094	10		**0.0002**
N	St.1, St.3		8.3616	0.2475	4		**0.0036**
	St.2, St.3		1.3299	0.4994	4		0.2865

The bold values indicate the results ≤ 0.05

*df* degree of freedom, *SS* sum squares, *MS* mean square, *Pseudo-F* Pseudo-*F* statistic, *P*_perm_ probability, *Perms* permutations, *P*_(MC)_ Monte Carlo probability, *M* Main Basin, *S* Southwestern Basin, *N* Northwestern Basin

The four GLMM models revealed a significant contribution of nine predictor variables for the infected fish model, but only a minimum of two predictor variables for the non-infected/infected ratio model (Table 6). However, the influence of biotic taxa had negative coefficients (− 0.12– −0.25), except for phytoplankton, and BMA, which were near zero (0.01). The TOM had the highest contribution to the fish models and ranged from −1.95 to −0.96, respectively, for the total fish and the infected fish with ectoparasites models. The regression *R*^2^ values ranged from 49 to 52%, except for the ratio model where *R*^2^ was only 8%. Meiofauna had the highest significant positive contribution to the infected fish (0.37), followed by a negligible direct contribution of phytoplankton and BMA in the same model.

**Table 6:** Results of forward selection GLMM analyses to predict best biotic diet regression relationships. Regressions based on square-root transformed data of four fish treatments (listed in Table 2) versus square-root transformed data of 11 predictor variables (listed in Table 4)

Predictor/effect	Total fish numbers			Non-infected individuals				
	*R*^2^ = 49%			*R*^2^ = 52%				
	*R*^2^_(adj)_ = 45%			*R*^2^_(adj)_ = 47%				
	*F*-ratio_(5, 69)_ = 13.28, *P* = **0.000**			*F*-ratio_(6,67)_ = 12.11, *P* = **0.000**				
	Coefficient	*T*-value	*P*	Coefficient	*T*-value		*P*	
Constant	6.26 (0.77)	8.13	**< 0.001**	6.46 (0.75)	8.57		**< 0.001**	
Meiofauna	*	*	*	0.03 (0.02)	1.59		0.116	
TOM%	−1.95 (0.30)	−6.52	**< 0.001**	−1.96 (0.31)	-6.25		**< 0.001**	
Harpacticoida	−0.06 (0.04)	1.79	0.078	*	*		*	
Ostracods	−0.12 (0.04)	−2.75	**0.008**	−0.13 (0.05)	−2.54		**0.014**	
Phytoplankton	0.009 (0.001)	6.08	**< 0.001**	0.01 (0.01)	6.45		**< 0.001**	
BMA	*	*	*	0.001 (0.01)	1.47		0.147	
Microbes	−0.09 (0.02)	−4.53	**< 0.001**	−0.15 (0.02)	−7.03		**< 0.001**	

	Infected individuals			Ratio (infected/non-infected)				
	*R*^2^ = 52%			*R*^2^ = 8%				
	*R*^2^_(adj)_ = 43.5%			*R*^2^_(adj)_ = 5.5%				
	*F*-ratio_(11,63)_ = 6.18, *P* = 0.000			*F*-ratio_(2,72)_ = 3.10, *P* = 0.051				
	Coefficient	*T*-value	*P*	Coefficient		*T*-value		*P*
Constant	2.45 (0.33)	7.40	**< 0.001**	0.45 (0.05)		8.30		**< 0.001**
Meiofauna	0.37 (0.12)	3.11	**0.003**	*		*		*
TOM%	−0.96 (0.14)	−6.70	**< 0.001**	*		*		*
Nematodes	−0.25 (0.09)	−2.71	**0.009**	*		*		*
Harpacticoida	−0.20 (0.05)	−3.69	**< 0.001**	*		*		*
Foraminifera	−0.15 (0.05)	−2.97	**0.004**	−0.02 (0.00)		−2.22		**0.029**
Ostracods	−0.13 (0.04)	−3.38	**0.001**	*		*		*
Turbellaria	−0.04 (0.03)	−1.38	0.173	*		*		*
Halacaridae	0.069 (0.07)	0.94	0.350	0.10 (0.05)		1.93		**0.057**
Phytoplankton	0.01(0.00)	5.52	**< 0.001**	*		*		*
BMA	0.00 (0.00)	3.01	**0.004**	*		*		*
Microbes	−0.02 (0.00)	−2.64	**0.010**	*		*		*

The bold numbers indicate significant values

*R*^*2*^ the statistic, *R*^2^_*adj*_ adjusted, *T-value T*-statistic, *P* probability at significant *α* level ≤ 0.05

*Symbol means omitted variable from the forward selection terms

**Fig. 4:**
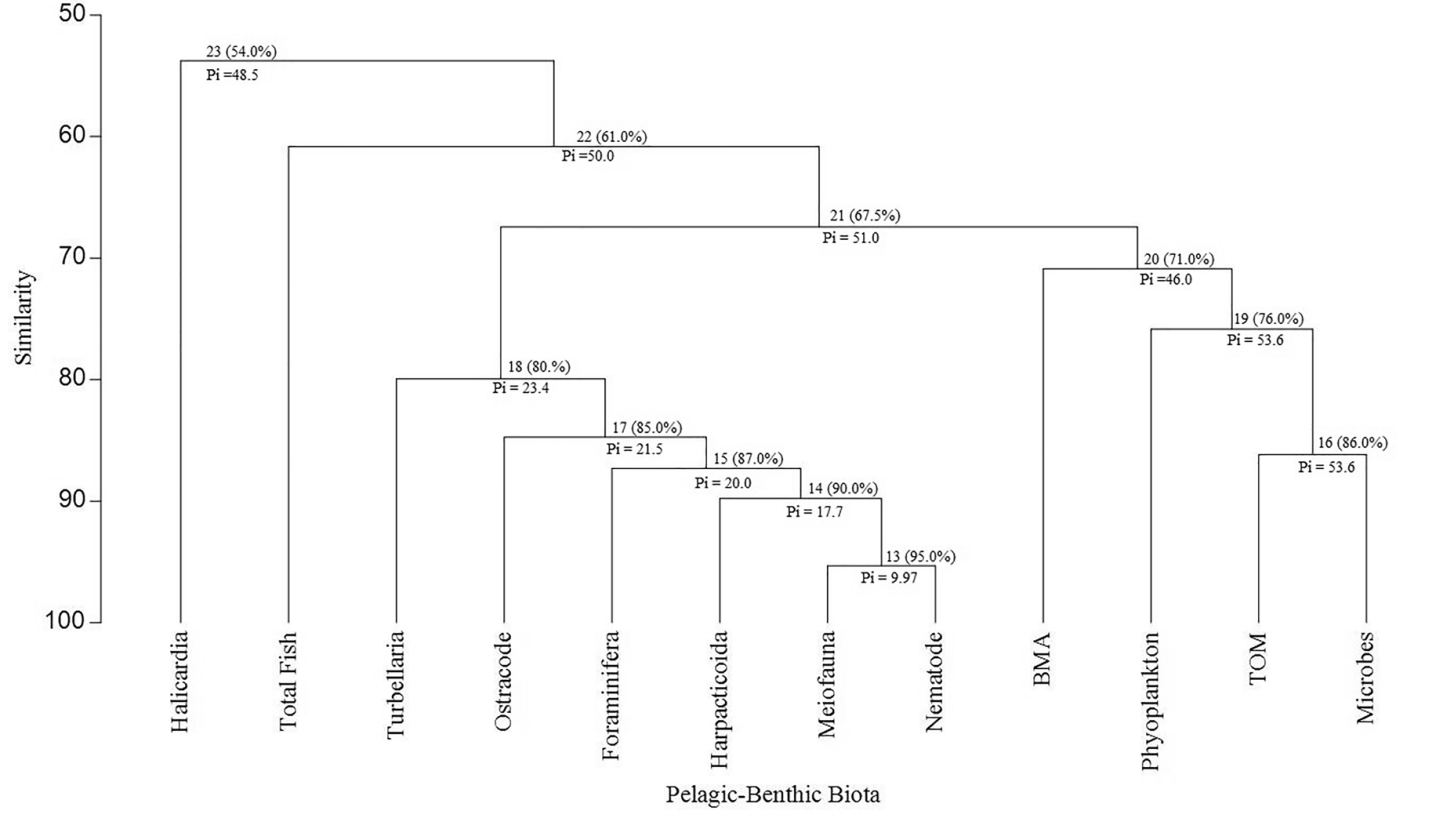
The hierarchical cluster analysis based on taxa levels and linkages at a significant level = 0.01. Similarity values in parentheses. Abbreviations: BMA, benthic microalgae; TOM%, percentage of total organic matter. All taxa were listed in Table 5

Dendrograms of the hierarchical cluster analysis (Fig. 4) showed 11 individual groups, forming four clusters, 16 significant ties (0.01), and their similarities decreased as the Pi statistic values increased, except for tie 19 (for phytoplankton similarity to TOM and microbes). The cluster on the right comprised phytoplankton, BMA, microbes, and TOM, comprising three pairs of ties, 16, 19, and 20, and are all primary producers. The similarities decreased from 86 to 71%, respectively, between ties 16 and 20. The clusters in the center represented sediment fauna that contained five ties; 13, 14, 15, 17, and 18, and their similarities percentage ranged from 95% with a tie between nematodes and meiofauna (13) to 80% between turbellaria and ostracods tie (18). The intermediate faunal cluster formed a significant linkage with the primary producers (> 67% similarity). The third cluster consisted of total fish numbers and linked significantly to linkage 21, forming a new one (22) with similarity and Pi values equal 61% and 50, respectively. The fourth cluster of Halacaridae (sea mites), a linkage 23, formed a link with the fish linkage 22 with the lowest similarity at 54%.

**Fig. 5:**
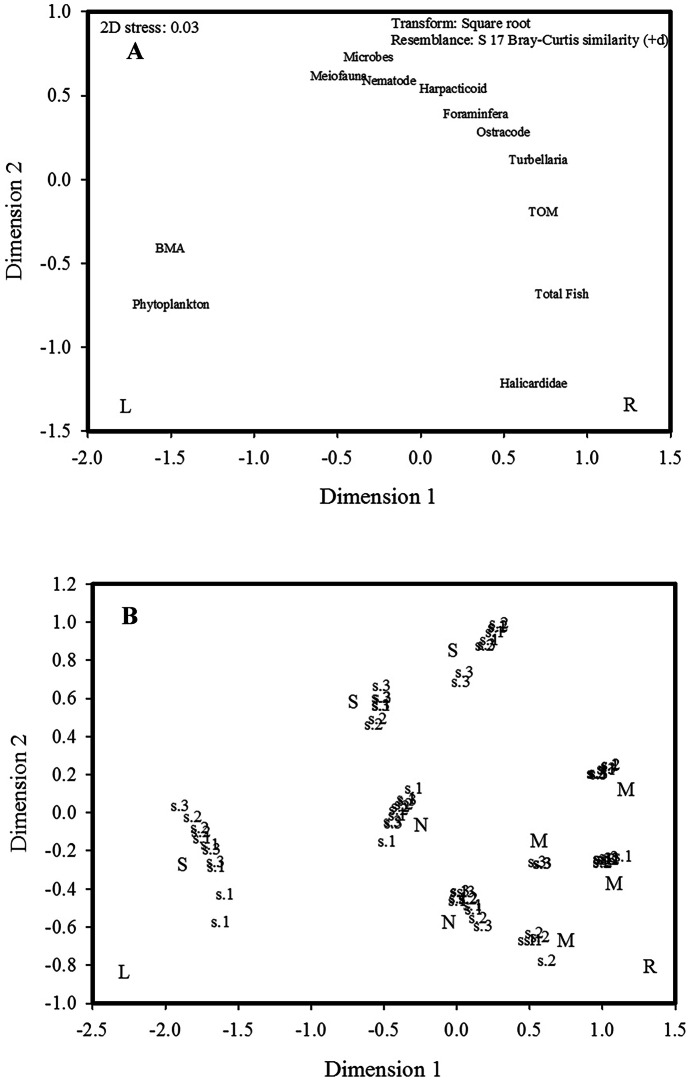
**A** The non-metric multidimensional scaling (*n*MDS) analysis for pelagic and benthic biota. Abbreviations: BMA, benthic microalgae; TOM%, percentage of total organic matter. **B** The *n*MDS analysis across the lake for multiple data groupings at each basin. Abbreviation: M, Main Basin; N, Northwestern Basin; S, Southwestern Basin. The numbers in the data labels indicate station and replicate

According to the results of the* n*MDS study (Fig. 5A), phytoplankton and BMA cluster close together on the left-hand side. The total number of fish is located at the bottom of the right-hand side, at equal distances from Halacaridae and TOM. Total meiofaunal, nematode, and microbial data were close to one another on the mid-top of the plot. Nematodes and TOM were arranged in a crescent shape among the other biota taxa. There were six distinct groupings among the basins (Fig. 5B). The M grouping was made up of four dispersed subgroupings evenly spaced between stations M1 and M3; all are on the right side of the plot. However, there was an overlap among stations’ replicates at the M. The S had three distinct subgroupings: S1, S2, and S3, which were distributed on the left, in the center, and somewhat to the right. In the middle, two prominent N groups formed. There is no overlap between replicated S and N stations. However, stations at each basin are clustering close to one another and in some cases; the deployments within stations are clustering.

Results of SIMPER analysis (Table 7) revealed similarities across each basin ranging from 84.60 (N) to 66.86% (S), and the range across the station groups (Gr.) varied between 61.26% (Gr.1) and 71.79% (Gr.3). The phytoplankton and BMA had the highest percentage contribution to the similarity across the M and N, and total meiofauna contributed 8.04% to the similarity at MB. The average dissimilarities between M vs. S, M vs. N, and S vs. N were 52.54%, 35.15%, and 39.60%, respectively, whereas values versus station groups fluctuated between ~ 41%, 40%, and 33%, respectively, for Gr.1 vs. Gr.2, Gr.1 vs. Gr.3, and Gr.2 vs. Gr.3. The contribution of the phytoplankton and BMA was responsible for the discrimination between basins (Table 6). The similarities among stations’ groups were higher than the dissimilarity values. The phytoplankton and BMA were essential contributors to similarities and dissimilarities among stations' groups, and meiofaunal contributed ~ 8.50% to the similarities within Gr.1 (stations 1) across all basins.

**Table 7:** Results of SIMPER analysis to test for species contribution across all basins and stations at a cut-off for low contribution (70%). Analysis based on Bray–Curtis similarity measures of square-root transformed data and the whole fish counts listed in Table 3

Average similarity of basin groups across all station groups						
Basin	Species	Av. abundance	Av. Sim	Sim./SD	Cont.%	Cum.%
M (82.59)	Phytoplankton	273.68	49.88	6.24	60.39	60.39
	BMA	162.62	22.89	2.13	27.71	88.11
S (66.86)	Phytoplankton	54.63	25.6	2.92	38.3	38.3
	BMA	90.42	19.61	0.88	29.34	67.63
	Meiofauna	13.03	5.37	1.73	8.04	75.67
N (84.60)	Phytoplankton	128.34	37.61	3.54	44.46	44.46
	BMA	78.61	25.94	8.4	30.66	75.11

Group M vs. S (average dissimilarity = 52.54)						
Species	Av. abundance	Av. abundance	Av. Diss	Diss./SD	Cont.%	Cum.%
Phytoplankton	273.68	54.63	32.17	3.81	61.24	61.24
BMA	162.62	90.42	16.15	1.43	30.74	91.98

Group M vs. N (average dissimilarity = 35.13)						
Phytoplankton	273.68	128.34	18.4	2.35	52.38	52.38
BMA	162.62	78.61	11.63	1.51	33.09	85.47

Group S vs. N (average dissimilarity = 39.60)						
Phytoplankton	54.63	128.34	15.76	1.75	39.79	39.79
BMA	90.42	78.61	14.72	2.5	37.18	76.97

Average similarity of station groups across all basin groups						
	Gr.1 (61.26)		Gr.2 (68.40)		Gr.3 (71.79)	
	St.1 (M, S, N)		St.2 (M, S, N)		St.3 (M, S, N)	
Species	Cont.%	Cum.%	Cont.%	Cum.%	Cont.%	Cum.%
Phytoplankton	42.82	42.82	42.3	42.3	46.52	46.52
BMA	24.09	66.91	33.36	75.65	38.62	85.13
Meiofauna	8.49	75.4	0	0	0	0

Average dissimilarity	Gr.1 vs.Gr.2 (40.72)		Gr.1 vs. Gr.3 (39.84(		Gr.2 vs.Gr.3 (33.10(	
Species	Cont.%	Cum.%	Cont.%	Cum.%	Cont.%	Cum.%
Phytoplankton	46.59	46.59	39.05	39.05	55.12	55.12
BMA	37.42	84.02	44.34	83.39	30.02	85.14

*Av.* average, *Sim.* similarity, *Sim./SD* similarity standard deviation, *Diss.* dissimilarity, *Cont. %* contribution %, *Cum. %* cumulative %, *Gr.* group, *M* Main Basin, *S* Southwestern Basin, *N* Northwestern Basin

## Discussion

The present study assessed the biological condition of the Lake Maruit ecosystem after restoration. Fish catch across the lake retained low number of species, consisting of three tilapia species: *Oreochromis niloticus*, *O. aureus*, and *Coptodon zillii*, and their distributions were similar among basins. Ectoparasites on fish had relatively low prevalence rates (3 to 15%). The helminthic Monogenea infested the two *Oreochromis* species, whereas the crustacean ectoparasites infected *C. zillii*. The total fish and the non-infected fish were correlated negatively with TOM, ostracods, and microbes. Surprisingly, the regression coefficients between the infected fish and most biota predictor data differed in sign and magnitude. The TOM concentration was related to fish abundance, but not phytoplankton and benthic microalgae, and there were no consistent pelagic–benthic linkages. The phytoplankton and benthic microalgae drove the similarity and the dissimilarity indices across the lake basins and stations, and meiofauna contributed to the similarity within the lake’s basins and nested stations. Comparisons with earlier studies prior to the restoration program (Table 1) revealed some evidence of restoration, such as low ectoparasites prevalence, similar fish and biota distribution, and a lack of eutrophication. The low number of catch species and abundance of fish and biota, the lack of correlations between fish and biota, and the inconsistent linkages between food web components suggest that the restoration is incomplete.n the linkageFisheries in Egyptian coastal lakes have suffered from overfishing, illegal fishing, and the deterioration of the water quality for many years, and this is likely a cause of decreased fish diversity (Mehanna, 2020). However, the species composition found in the present study (i.e., 3 species) was low relative to the 25 and 11 species found in Lake El Borollus and Qarun Lake, respectively (El-Serafy et al., 2014; Younis, 2018). Our findings might be attributed to the synergistic effects of the restoration and an accidental oil leak from the Al-Amerya and Pertoject petroleum companies. Mechanical and hydraulic dredging of the aquatic ecosystem increases fish mortality, impacts physiology, and changes fish behavior (Wenger et al., 2017). The polycyclic aromatic hydrocarbon concentrations were one to two orders of magnitude higher at Lake Maruit than in other estuaries worldwide (Barakat et al., 2010). Moreover, according to the General Authority for Fish Resources Development (GAFRD), the total fish production from Egypt’s natural lakes has dramatically declined from 43% by the end of the 1990s to 19% in 2018 (Mehanna, 2022).

The ectoparasites infection rate in the current study was 3% (parasitic-copepod) and 15% (Monogenea), which is a relatively low infestation rate compared to the 57% rate at Qarun Lake (Mehanna, 2020). El-Rashidy (unpublished data) examined tilapia fish for parasitic copepods from Lake Maruit in 2000 before lake restoration and detected a 15.5% prevalence of *E. lizae* on tilapia species. Nofal and Abdel-Latif (2017) recorded an 76% infection rate of Monogenea in Lake Manzala, accompanied by massive fish death due to deterioration of water quality. The infection rate found in Lake Maruit lies between the frequent and irregular Monogenea infection rate according to Koniyo et al. (2020) and a low copepod prevalence (Klimpel et al., 2006). Mitwally (2015) and Shreadah et al. (2020) documented noticeable improvements in Lake Maruit environmental conditions, except for phosphorous loading, which could be responsible for the infestation rate decline. Low ectoparasites prevalence is a good sign of lake rehabilitation after the restoration process.

Heterogeneous distributions of benthic biota are common in disturbed versus undisturbed environments (Pan et al., 2000; Solar et al., 2015). So, the lack of significant variations among basins found here could be a good indicator of the restoration success. All the biota responded to the environmental conditions in a similar way, and environmental conditions were suitable for pelagic and benthic biota to survive. Most physicochemical and sedimentological parameters were improved after the restoration program (Mitwally, 2015; Shreadah et al., 2020). However, the causes of the relatively low diversity of benthic biota in Lake Maruit are probably related to long-term exposure to disturbance, and these biotas require longer periods to recover. It can take a long time before biota composition can recover from disturbance in ecosystems according to Janse et al. (2015). High variability at the smaller station-scale (Tables 3, 4, and 5) agreed with findings by Mitwally and Abada (2008) indicating that small-scale heterogeneous distributions are due to the local biological interaction and physical processes, which could attenuate among-basin variability.

Aquatic ecosystems contain many complex trophic links (Gallardo et al., 2016) but the multiple linear regression (Table 6) indicates that any specific taxa is potentially part of the fish diet in Lake Maruit. The lack of strong links could be related to the irregularity of fish feeding patterns and opportunistic feeding habits. Tilapia species diets include fauna and flora (El-Sayed, 2019). However, the inverse relationship between sediment–biota and fish may reflect their different responses to TOM as a proxy for allochthonous sources flowing into Lake Maruit. The TOM concentration on average (Table 2) was ~ 7 times higher than that at El-Mex Bay (1.43%), according to Salem et al. (2013). The allochthonous organic matter effects fish production and food web productivity (Craig et al., 2015), probably due to the attenuation of light penetration, as suggested by Karlsson et al. (2009). Mitwally (2015) documented a positive response between meiofauna and organic matter, whereas fish abundance had a negative linear regression with TOM in Lake Maruit (Table 6). Fish and benthic fauna respond differently to anthropogenic stressors in shallow ecosystems (Snickars et al., 2014). There is a linear regression link between the numbers of biota and the fish with ectoparasite infections (Table 6), and this is likely because parasites alter their host’s behavior and may have indirectly mediated the relationships between predators and prey (Forrester et al., 2019; Grutter et al., 2011). Johnson (2001) discovered a change in fish diet following parasite infection, although the positive association between total meiofauna and infected fish suggests that fish may switch diets as an indirect effect of parasite infestation. It is necessary to conduct more research on the feeding strategy and the impact of ectoparasites on fish behavior and community structure.

The food web in aquatic habitats is complex and has intricate connections (Woodward, 2009). However, the Lake Maruit food web consisted of four simple groups (Fig. 4), probably due to the absence of some trophic links, such as macrofauna and zooplankton. Thompson et al. (2012) concluded that some highly resolved, very comprehensive food webs still have limitations, as they ignored microbial taxa and soft-bodied meiofaunal taxa because obtaining the data is time-consuming and expensive. The presence of the four components of primary producers (i.e., phytoplankton, BMA, microbes, and organic matter) indicates that Lake Maruit is a grazing and detritus-based ecosystem. The intermediate faunal cluster (Fig. 4) highlights the importance of meiofauna as a central trophic linkage, not a dead-end, which agrees with Schmid-Araya et al. (2002). The high number of linkages (22) between fish, meiofauna, and primary producers (Fig. 4) reflects that tilapia species likely have opportunistic feeding modes and that meiofaunal organisms are likely part of the diet for fish in Lake Maruit, as Schmid-Araya et al. (2016) concluded. However, Halacaridae (Fig. 5) was the only biota that had a direct link with fish occupying the top of the food chain, which is an inconsistent finding in that it is not found in other natural ecosystems (Peel et al., 2019). The causes of this contradiction may be connected to the anthropogenic stressors impacting Lake Maruit that caused lake size shrinkage (from 200 to 63 km^2^) and the engineered restoration, as the natural disruption and human-induced changes modified the food web (Tunney et al., 2012). However, it is also possible that the fish and Halacaridae are simply correlated without causation or have similar diets. Further biomass investigations beyond the abundance data are recommended in future studies for a better understanding of food web connectivity.

Phytoplankton and BMA comprised a bulk of the *O. niloticus* diet (Dempster et al., 1993), detritus represented ~ 45% of *O. aureus* (Al-Wan & Mohamed, 2019), and plant tissue was dominant in *C. zillii* stomach (Shalloof et al., 2020). Our findings of the correlation between TOM, sediment biota, and fish, and between fish, phytoplankton, and BMA (Fig. 5A) could indicate that Lake Maruit is a detritus-driven ecosystem. The high TOM concentration drives the relationship between pelagic and benthic habitats, as it reduced light, diminishing fish vision, and decreased the accessibility of sediment fauna as fish prey. The Halacaridae’s size ranged from 0.75 to 0.90 mm (Proctor, 2004), and this may allow the fish’s vision to hunt them easily. It is known that the larger the prey size, the greater the fish’s ability to locate and eat them (Wetzel, 2001). Many studies documented fish feeding on Halacaridae (Luxton, 1990; Mcmahon et al., 2005), meiofaunal taxa (Ptatscheck et al., 2020), and the intensive feeding of *O. niloticus* in the River Nile, Egypt, on nematodes (Abada et al., 2017). The intermediate trophic link of zooplankton and the low coefficients (Table 6) between phytoplankton, BMA, and fish indicates a lack of a strong trophic link among these groups.

The multiple subset groups in each basin (Fig. 5B) are likely the result of the different modes of biota lifestyle and dispersion. Four subgroupings at the M basin revealed the active movement of pelagic fish, horizontal phytoplankton passive movement, and sediment-associated biota with limited vertical passive migrations of meiofauna. However, two subgroups at N and S suggested the unsuitability of these basins to the active fish movement, probably due to smaller surface area and shallow depths. The dynamic dispersal modes enable the fish to move freely and select the most suitable environmental conditions, whereas the passive phytoplankton dispersers are limited by wind-induced currents (Lansac-Toha et al., 2019). The different dispersal modes of biota drove the compositional dissimilarity within a lentic ecosystem (Oikonomou & Stefanidis, 2020). The similarity within MB data suggested obvious linkages between pelagic and benthic biota, and the overlapping indicated the high basins similarity.

The relatively high values of similarity percentages among basins compared to those within station groups in the ANOVAs (Tables 3 and 4) and the *n*MDS (Fig. 5B) indicate that heterogeneity is higher in small spatial scales than larger spatial scales. This is probably due to the variable local environmental factors at each site. Hawkins et al. (2000) attributed the significant differences in fish and fauna within small distances to local environmental habitat features of each location and commented that similarities among sites declined as a function of the distances between them.

Phytoplankton and BMA were responsible for the high similarity within the MB and NW (Table 7), probably due to passive dispersion being the driver of phytoplankton distribution (Beisner et al., 2006). However, the higher average abundance of BMA abundance compared to phytoplankton abundance at the S could be the reason for the increase in total meiofauna contribution to the similarity (Table 7), as BMA is a favorable meiofaunal diet (Mitwally et al., 2004; Montagna, 1984). The synergetic factors within each basin at Lake Maruit were responsible for the meiofauna response to the environmental conditions (Mitwally, 2015).

Comparisons with earlier studies before restoration (Abdallah, 2011; Khalil, 1998) revealed some temporary signs of rehabilitation, as eutrophication was not indicated by the decline in phytoplankton abundance, which was three times (Table 2) lower than that recorded in autumn 2004 (Hussein & Gharib, 2012). Microbial counts were low relative to those found by Hassan and El-Rayis (2018), which is an indicator of sewage discharge reduction. The high phosphorous concentration (Mitwally, 2015; Shreadah et al., 2020) probably was the factor that masked the long-term restoration effectiveness, as an increase in chlorophyll concentration and bacteria was detected in 2016 (Abd El-alkhoris et al., 2020; Abdelfattah et al., 2017; El Zokm et al., 2018) (Table 1). Meiofauna abundance at Lake Maruit was slightly lower (Mitwally, 2015) than that at the least polluted El Borollus Lake (Mitwally & Abada, 2008), indicating meiofaunal tolerance to disturbance due to its broad range of groups (Schratzberger & Warwick, 1999), rapid re-colonization, and ability to migrate down the sediment (Schratzberger et al., 2000). The macrofaunal size fraction was dominated by broken shells and fragmented bodies of three groups after restoration (Mitwally, 2015) compared to 11–12 species before restoration (Khalil & Koussa, 2013a; Khalil et al., 2016), indicating macrofaunal sensitivity to the mechanical restoration processes. However, we excluded macrofauna and zooplankton from the current study because of the prevalence of dead macrofauna bodies and the relatively empty zooplankton samples after restoration versus Khalil and Koussa (2013b) before restoration. A low ectoparasite infestation rate was an indicator of restoration effectiveness. However, the drop in pelagic and benthic biota abundances, lack of trophic correlations, and inconsistent linkages among trophic groups is likely due to mechanical effects of restoration construction, such as dredging impacts aquatic life (Chen et al., 2021). Further monitoring over time and seasons is needed to assess biota recovery after restoration and to evaluate long-term change.

In the current study, pelagic and benthic biota interactions could be used as bioindicators in monitoring and assessment programs for aquatic habitats. Some evidence indicates stressed vs. unstressed ecosystems. The stressed ecosystem suffered from low fish species and other biota abundances compared to its earlier history or surrounding areas. The inconsistency in the food web and the lack of direct relationships between prey and predators are other bioindicators of disturbed ecosystems. Bioindicators of the rehabilitated habitat’s unstressed condition are a low prevalence of ectoparasites on fish and the absence of heterogeneous distribution of different investigated biota.

## Conclusion

The current study assessed the Lake Maruit, Egypt, after the restoration of long-term deterioration. The low prevalence of ectoparasites on fish and the high similarity of pelagic–benthic biota distribution across lake basins are positive indicators for lake rehabilitation after the restoration. However, the inconsistent links in the food web and the high organic matter that correlate with different biota could be due to the anthropogenic stressor. The large size of Halacaridae enables fish to locate and prey up on them and could be the cause of their positive correlation. Our results concluded that there is some evidence of the effectiveness of the restoration program at Lake Maruit. However, others could indicate that Lake Maruit suffered from degradation in 2012.

## Data Availability

The data that support the findings of this study are available from the corresponding author, upon reasonable request.
